# Estimation of transmission parameters of a fluoroquinolone-resistant *Escherichia coli *strain between pigs in experimental conditions

**DOI:** 10.1186/1297-9716-42-44

**Published:** 2011-03-02

**Authors:** Mathieu Andraud, Nicolas Rose, Michel Laurentie, Pascal Sanders, Aurélie Le Roux, Roland Cariolet, Claire Chauvin, Eric Jouy

**Affiliations:** 1 Anses, Fougères laboratory, BP 90203, F-35302 Fougères Cedex, France; 2 Centre for Health Economics Research and Modeling Infectious Diseases (CHERMID), Vaccine & Infectious Disease Institute (VAXINFECTIO), University of Antwerp, Antwerp 2610, Belgium; 3 Anses, Ploufragan Plouzané laboratory, BP 53, F-22440 Ploufragan, France

## Abstract

Antimicrobial resistance is of primary importance regarding public and animal health issues. Persistence and spread of resistant strains within a population contribute to the maintenance of a reservoir and lead to treatment failure. An experimental trial was carried out to study the horizontal transmission of a fluoroquinolone-resistant *Escherichia coli *strain from inoculated to naïve pigs. All naïve contact pigs had positive counts of fluoroquinolone-resistant *E. coli *after only two days of contact. Moreover, re-infections of inoculated pigs caused by newly contaminated animals were suspected. A maximum likelihood method, based on a susceptible-infectious-susceptible (SIS) model, was used to determine the transmission parameters. Two transmission levels were identified depending on the quantity of bacteria shed by infected individuals: (i) low-shedders with bacterial counts of resistant *E. coli *in the faeces between 5*10^3 ^and 10^6 ^CFU/g (*β*_*L *_= 0.41 [0.27; 0.62]), (ii) high shedders with bacterial counts above 10^6 ^CFU/g (*β*_*H *_= 0.98 [0.59; 1.62]). Hence, transmission between animals could be pivotal in explaining the persistence of resistant bacteria within pig herds.

## Introduction

Antimicrobial resistance is an important concern in animal health. Antimicrobial treatment causes selective pressure that can trigger the selection of resistant bacteria [1]. The selection, persistence and spread of such strains within a population is often puzzling and can lead to failures of the usual treatments against infections [2]. Moreover, administration of antimicrobial agents used in human medicine to production animals, such as fluoroquinolones, could lead to the emergence of public health issues due to the persistence and dissemination of resistant bacteria [3]. Although surveillance of resistance in zoonotic bacteria has a direct implication on public health policies, monitoring bacteria from the commensal flora, such as *Escherichia coli *strains, remains a good indicator of the resistance pattern within a population. Knowledge of the resistance pattern in such commensal bacteria is pivotal because of the risk of transmission of resistant genes from non-pathogenic to zoonotic bacteria [4].

Quinolones are widely used in pig production, mainly to control urinary tract infections in sows [5]. Belloc et al. [6] observed that quinolone treatment caused a strong selective pressure in the *E. coli *population of the treated sows and their piglets. In this study, the treatment was routinely administered to pregnant sows around farrowing-time. Even though the effects were transitory, the faecal flora of the piglets was clearly modified by the treatment of their dams, leading to a dramatic increase in the within-host proportion of resistant *E. coli*. The long-term impact of such routine practices of antimicrobial use is unknown, but enrichment of the resistant flora with duration of use is highly suspected [7]. Studies of the long term ecological evolution within a population require a mathematical modelling approach [8,9].

To date only a few epidemiological models have been developed to study the spread of infectious agents within pig-production units [1,10-12]. To the best of our knowledge, only one of these modelling approaches focussed on the impact of antimicrobial consumption on the emergence and/or transmission of resistant strains within a finisher pig herd [13]. This study highlighted the need for a modelling framework which incorporates both current knowledge of the epidemiology of antimicrobial resistance in animals and the key factors which can be used to characterize within-herd dynamics of resistant bacteria under the influence of antimicrobial pressure. Knowledge of the transmission dynamics of resistant bacteria would improve the understanding of the influence of on-site farming practices (drug usage, infection control) on the dynamics of resistant bacteria in animals and could also form the basis for a release assessment (component of a risk assessment). Abatih et al. [13] used a deterministic SIS (Susceptible-Infectious-Susceptible) model in which infectious individuals were divided into two sub-groups (*I*_*I *_and *I*_*R*_) according to their levels of susceptibility to antimicrobial agents. The authors concluded that the persistence of resistant bacteria within the finishers was strongly related to the transmission parameter values [13].

Transmission rates (*β*) are key parameters of epidemiological models. However, estimation of these parameters often presents a challenge since, in many infectious diseases, only the onset of clinical symptoms or the final outcome of the disease can be observed in field conditions. Experimental transmission trials provide a useful tool for assessing infection dynamics in a controlled environment [14]. Different estimation methods have been used to analyse results from transmission experiments: maximum likelihood estimation (MLE, [15-19]), generalised linear model (GLM, [20-22]) or Becker's martingale formulae based on the final size of the epidemics [14,23,24]. The aim in these studies was to quantify the transmission of pathogenic infectious agents (viral or bacterial). However, to the best of our knowledge, none of these experimental studies focussed on the transmission of resistant bacterial strains.

The aim in the present work was to study the horizontal transmission of a fluoroquinolone-resistant *E. coli *strain between pigs. For this purpose, transmission experiments were carried out by focussing on the transmission of an in vitro selected *E. coli *from inoculated to naïve specific pathogen free (SPF) piglets. Transmission parameters were estimated by applying a maximum likelihood method which accounted for different transmission levels related to the amounts of resistant bacteria recovered from the individual faeces of infected animals.

## Materials and methods

### Experimental design

**Figure 1:**
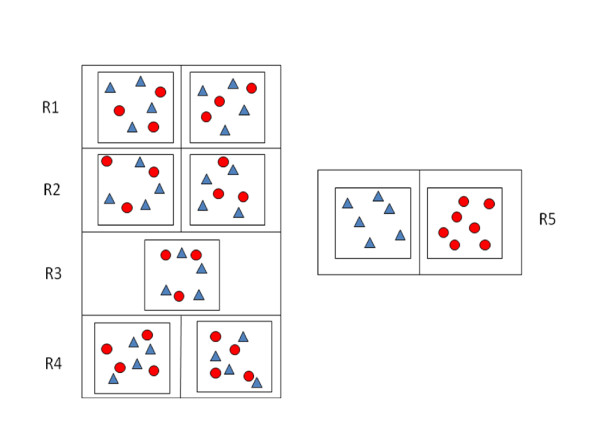
**Experimental design for quantification of fluoroquinolone-resistant *E. coli *transmission from inoculated (circles) to naïve specific pathogen free (SPF) pigs (triangles)**.

The experiment was conducted in our air-filtered level 3 biosecurity-facilities. Five independent rooms were used (R1 to R5) with one or two pens per room (Figure 1). Pigs were housed on flat decks, with a fully slatted floor one metre above the floor of the facility. Sixty-four Large-White SPF pigs, aged seven weeks at the beginning of the experiment, were used in this study. Animals were weaned three weeks before the experiment and received non-supplemented feed. No antimicrobial treatment was administered either before the start or during the course of the experiment. Six pigs were kept as negative controls and 7 as positive inoculated controls (R5). The 51 remaining pigs were randomly assigned to 5 groups of 7 pigs (3 inoculated and 4 naïve SPF pigs) and 2 groups of 8 pigs (4 inoculated and 4 naïve SPF pigs). Each group was conducted separately and between-group transmission was avoided by having a solid partition between pens. Inoculations were performed orally, by successively administering 10 mL of a bacterial suspension (10^9 ^CFU/mL), on the day prior to contact with SPF pigs and on the contact-day. Contact was instigated by mixing the inoculated and naïve pigs about four hours after the second inoculation.

The bacterial suspension consisted of a ciprofloxacin-resistant *E. coli *in vitro selected from the faecal flora of a fully susceptible *E. coli *strain isolated from Specific Pathogen Free (SPF) pigs of the same herd. The selection of *E. coli *with chromosomal mutations was performed by spreading a large amount of inoculum (about 10^9 ^CFU) onto plates supplemented with increasing concentrations of ciprofloxacin (Sigma/Fluka, St-Quentin-Fallavier, France). The minimum inhibitory concentration of ciprofloxacin for the selected resistant strain was 1.5 mg/L (E-test, bioMérieux, Marcy-l'Étoile, France). The suspension was administered using a soft sterile catheter which was gently but deeply introduced into the oesophagus to prevent the inoculum from flowing back and further contaminating the environment.

All pigs were monitored daily by taking faecal samples from the day preceding inoculation until 4 days post-inoculation and then every 2 days until day 10. Clinical signs were also recorded daily and the pigs were weighed once a week. Bacterial counts were measured on 1 g of faeces using serial 10-fold dilutions. One hundred microliters of each dilution were plated on chromogenic agar (Chromocult Agar ES, Merck/VWR, Strasbourg, France) supplemented with 0.5 mg/L of ciprofloxacin (Sigma/Fluka, St-Quentin-Fallavier, France). The plates were incubated at 37°C for 24 h then the colonies were counted. This method allowed quantification of the concentrations of inoculated *E. coli *greater than or equal to 10^2 ^CFU/g of faeces.

The experiment was ended two weeks after inoculation. Euthanasia was carried out by anaesthesia with an intravenous injection of 1g/50 kg liveweight of Nesdonal^® ^(Merial, Lyon, France) followed by exsanguination. The following organs were examined macroscopically immediately after killing: lungs, tonsils, heart, kidneys, thymus, lymph nodes (inguinal, mesenteric, tracheo-bronchial) and the different parts of the digestive tract (oesophagus, stomach, duodenum, jejunum, ileum, colon and caecum). When macroscopic lesions were suspected, samples were taken from the tissues for histopathological examination.

The experiment was performed in accordance with EU and French regulations on animal welfare in experimentation. The protocol was approved by the AFSSA/ENVA/UPEC ethical committee.

### Comparison of bacterial counts between groups

An analysis of variance was used to compare bacterial counts according to animal status (contact or inoculated pigs) and group size (7 or 8 pigs per pen). These comparisons were performed on the bacteriological results obtained at the start (D_1_) and end (D_10_) of the experiment (*lm *function, R software [25]).

### Quantification of transmission

Based on the bacteriological results, a stochastic SIS (Susceptible-Infectious-Susceptible) model was used to estimate the transmission parameters. As in a classical SIR (Susceptible-Infectious-Removed) model the rate at which susceptible animals (S) were infected is given by *βSI/N*, where *β *is the transmission parameter, defined as the mean number of new infections caused by a typical infectious individual per unit of time [26,27], while *I *and *N *represent the number of infectious and the total number of individuals, respectively. Pigs were classified as susceptible or infectious according to the quantities of resistant bacteria recorded in individual faecal samples. Changes in individual status observed between two sampling dates, either from susceptible to infectious or reciprocally, were considered to occur at the mid-time interval.

Individuals were considered susceptible when the quantity of fluoroquinolone-resistant *E. coli *was below a threshold level (*T*_1_), fixed at 5*10^3 ^CFU/g in accordance with literature data [28]. Individuals with bacterial counts above this threshold quantity were considered as infectious, i.e. were considered able to transmit resistant bacteria to susceptible pigs. Three hypotheses (H1, H2, and H3) were proposed regarding the ability of infectious animals to transmit resistant bacteria to susceptible ones, depending on the amount of resistant bacteria in individual faecal samples:

- H1. Equal transmission rate for all shedding pigs with bacterial counts above the threshold value *T*_1 _(5*10^3 ^CFU/g).

- H2. Two transmission levels according to the bacterial counts: Pigs shedding between 5*10^3 ^and 10^6 ^CFU/g of resistant *E. coli *were considered as low shedders (*I*_*L*_) as compared with pigs shedding more than 10^6 ^CFU/g of faeces (*I*_*H*_).

- H3. Three transmission levels according to the bacterial counts. An intermediate shedding level was included: pigs shedding between 5*10^3 ^and 10^5 ^CFU/g of resistant *E. coli *were considered as low shedders (*I*_*L*_); but if the bacterial counts ranged between 10^5 ^and 10^6 ^CFU/g, the pigs were classified as moderate shedders (*I*_*M*_), whereas, in high shedders, as with hypothesis 2, the bacterial counts exceeded 10^6 ^CFU/g (*I*_*H*_).

These hypotheses led to different models of increasing complexity which could be used to study the relationship between the shedding pattern and transmission potential of infectious pigs. However, the model obtained under the third hypothesis (H3) could be considered as the full model with three transmission parameters: *β*_*L*_, *β*_*M *_and *β*_*H*_, the second hypothesis (H2) being a simplification of this model in which *β*_*L *_= *β*_*M*_, and the first hypothesis (H1) derived from the latter by assigning equal values to all the transmission parameters (*β*_*L *_= *β*_*M *_= *β*_*H*_).

The maximum likelihood method, based on a stochastic SIS (Susceptible-Infectious-Susceptible) model, was used to estimate the transmission parameters. It follows from the model that the probability *q *that a single susceptible pig escapes infection during a time interval of *d *is equal to *q *= exp(-*d*(∑_*i *_*β*_*i *_π_*i*_)), where *i *represents the transmission level of infectious pigs (*i*∈{*L*, *M*, *H*} for Low-, Moderate- and High-shedders). In this equation, π_*i *_are the proportions of infectious animals in each shedding class, and *β*_*i *_the corresponding transmission parameter. *p *= 1 - *q *is then the probability that a susceptible individual will be infected and the number of new infections follows a Binomial distribution *C ~ Bin*(*S, p*) with *S *the number of susceptible animals in contact. The log-likelihood for this Binomial distribution is $\log\left(L\left(\beta_{L},\beta_{M},\beta_{H}\right)\right)=\sum_{j}\left[C_{j}\log\left(\text{exp}\left\{d_{j}\left(\sum_{i}\beta_{i}\pi_{i}\right)\right\}\;-\;1\right)\;-\;S_{j}d_{j}\left(\sum_{i}\beta_{i}\pi_{i}\right)\right]$ with *S_j _*and *C_j _*the number of susceptible animals and cases at each sampling interval *j *respectively, *d_j _*the duration of the interval between two sampling times, and where $\log\left(\begin{array}{l} S_{j}\\ C_{j} \end{array}\right)$ is omitted because it plays no role in the calculations. The different *β *parameters were estimated using maximization of the log-likelihood with *nlm *function in *R *software (Version 2.6.0., [25]). Confidence intervals were determined using the inverse of the Hessian matrix (variance/covariance matrix) of parameter estimates, also provided by *nlm *function in *R *software.

## Results

### Transmission results

**Figure 2:**
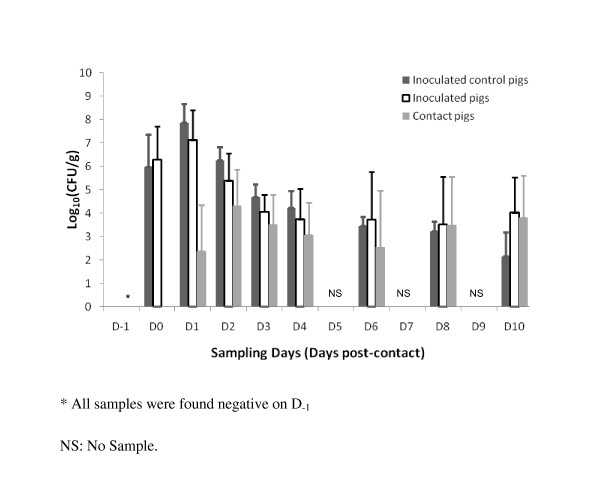
**Mean number and standard deviation (error bars) of fluoroquinolone-resistant *E. coli *in faeces of inoculated (*n *= 23), inoculated control (*n *= 7) and contact pigs (*n *= 28)**.

No clinical sign was observed during the experiment and the daily weight gain (DWG) was similar in challenged groups (Figure 1, R1 to R4), control pigs (R5) and in individuals from the contact groups (data not shown). No resistant *E. coli *was isolated either before inoculation of pigs in the contact groups or in the negative control group throughout the entire experiment. All animals in the inoculated control group had positive resistant bacterial counts on the contact-day (D_0_) after which these counts declined steadily from D_1 _to the end of the experiment (D_10_) (Figure 2). All inoculated pigs in the contact groups had positive resistant bacterial counts on D_0_, i.e. one day after their first inoculation. Individual bacterial counts in inoculated pigs increased until day 1 post-contact, ranging between 4.66 and 8.96 log_10_(CFU/g). The bacterial counts of the inoculated pigs then decreased over a period of 3 to 5 days. An inverse trend, with an increase in bacterial counts, was observed in 13 of the 23 inoculated pigs between day 4 and day 10 post-contact. This was probably due to the infectious pressure exerted by contaminated contact pigs as such a tendency was not observed in the inoculated control group. A similar pattern was observed in the contact groups with a lag of 1 to 2 days. Nineteen out of 28 contact pigs had positive bacterial counts on day 1 post-contact and all pigs in the contact groups were found positive on day 2 post-contact. An overall decrease in bacterial counts was observed until the 6^th ^day post-contact. Similar transmission behaviours were observed in all the pens. No significant difference was found in individual bacterial counts according to the number of pigs per pen (5 and 2 trials involving 3 or 4 inoculated pigs respectively, in contact with 4 naïve pigs) either in inoculated or in contact pigs (Table 1). Equivalent bacterial counts were recorded on days 8 and 10 post-contact in both the inoculated and contact groups (*p *= 0.66) (Figure 2).

**Table 1:** Comparison of mean bacterial counts at days 1 and 10 post contact between inoculated (I) and contact (C) pigs and between experimental settings (3I × 4C versus 4I × 4C trials).

	Mean Bacterial Count (Log_10_(CFU/g))					
**Time of comparison (day post-contact)**	**1**			**10**		

	**Estimate**	**SD**	***P*-value**	**Estimate**	**SD**	***P*-value**

Intercept	2.45	0.37	< 0.001	3.84	0.38	< 0.001
Group size* (*n *= 8/*n *= 7)	-0.27	0.70	0.70	-0.11	0.70	0.87
Status (inoculated/contact)	4.34	0.57	< 0.001	-0.08	0.57	0.89
Interaction (Group size*Status)	1.22	1.01	0.24	0.84	1.01	0.41

*n: number of animals per pen in the experimental settings. *n *= 7: 3 inoculated and 4 naïve pigs, *n *= 8: 4 inoculated pigs in contact with 4 naïve pigs.

During the experiment, the resistant bacterial counts in at least one sample from 12 contact and 8 inoculated pigs was below the detection limit of the method, but the resistant strain could not be detected on D_10 _in only three pigs. All but 5 inoculated pigs (18/23) had bacterial counts over 10^6 ^CFU/g on D_0 _and D_1_. Only 7 of these high-shedders still showed high shedding levels on day 2 post-contact. The moderate shedding level, between 10^5 ^and 10^6 ^CFU/g, was found to be a transient phase between low- and high-shedding.

### Transmission parameter estimates

Transmission parameters differed according to the assumption made about the shedding pattern (Table 2). As all three models were nested, the Akaike information criterion (AIC) was applied for model selection. The AIC values were very close whatever the underlying hypothesis (H1, H2 or H3). The lowest AIC was obtained with the second hypothesis (H2, *AIC *= 117.85) in which two transmission rates were considered. The estimated transmission of low-shedders, in this model, was 2.5 times less efficient than that of high-shedders (*β*_*L *_= 0.41 [0.27; 0.62], *β*_*H *_= 0.98 [0.59; 1.62]). These estimates were not modified by introducing an intermediate transmission level (H3), corresponding to pigs shedding more than 10^5 ^and less than 10^6 ^CFU/g. *β*_*M *_was very close to the *β*_*L *_estimate with a larger confidence interval due to the limited number of animals in this class (*β*_*M *_= 0.42 [0.15; 1.19]).

**Table 2:** Transmission parameter estimates (and their 95% confidence intervals).

Hypothesis*	Parameters			AIC
		
	*β*_*L *_(CI)	*β*_*M *_(CI)	*β*_*H *_(CI)	
H1	0.55 (0.40; 0.74)	-	-	121.00
H2	0.41 (0.27; 0.62)	-	0.98 (0.59; 1.62)	117.85
H3	0.41 (0.23; 0.71)	0.42 (0.15; 1.19)	0.98 (0.59; 1.62)	119.85

* Hypothesis on the relationship between bacterial counts and transmission efficiency:

H1. Equal transmission parameters (*β*_*L*_) for all shedding pigs with bacterial counts above the threshold value (5*10^3 ^CFU/g).

H2. Two transmission parameters according to bacterial counts: *β*_*L *_for low-shedders (bacterial counts between 5*10^3 ^and 10^6 ^CFU/g) and *β*_*H *_for high-shedders (bacterial counts above 10^6 ^CFU/g).

H3. Three transmission parameters according to bacterial counts: *β*_*L *_for low-shedders (bacterial counts between 5*10^3 ^and 10^5 ^CFU/g), *β*_*M *_for moderate-shedders (bacterial counts between 10^5 ^and 10^6 ^CFU/g), and *β*_*H *_for high-shedders (bacterial counts above 10^6 ^CFU/g).

## Discussion

The aim of this study was to assess the horizontal transmission of a fluoroquinolone-resistant *E. coli *within a swine population. Although the emergence and selection of antimicrobial-resistance can be linked with antimicrobial consumption [29], the transmission of resistant bacteria between individuals could be a pivotal factor governing persistence within a pig herd. Such transmission may occur in swine herds when mixing pigs which have been treated or not, or litters derived from treated and non-treated sows. As very few observations and no parameter estimates were available for the passive horizontal transmission of resistant commensal bacteria between animals [13], an experimental trial was designed to study the transmission of fluoroquinolone-resistant *E. coli *from inoculated to naïve pigs, in the absence of selective pressure.

When studying a natural infection, the use of contact animals, rather than artificially inoculated animals with a presumed higher infectiousness, has been recommended to start the infection chain under study [30]. Only artificially inoculated pigs were used in the present experiment, in order to limit the number of pigs involved. However, the amounts of resistant bacteria shed by inoculated pigs on the day of contact were within the range observed in treated animals [31] or in piglets born to treated sows [6]. In addition, no significant difference was found between the amounts of bacteria shed by inoculated and contact pigs. The present results can therefore be considered as realistic and representative of the transmission pattern occurring under field conditions. Moreover, to date no plasmid-mediated quinolone resistance has been reported in *E. coli *isolates from pigs in France, so this observation can be considered as consistent with the current epidemiological on-farm situation.

The experimental design was developed to mimic a situation found under field conditions and represented the mixing of piglets from different litters with heterogeneous resistance patterns. As suggested by Kroese and de Jong [14], the numbers of inoculated and contact pigs were equivalent in each experimental trial, with slightly fewer inoculated pigs in 5 pens (3 inoculated versus 4 contact animals) which did not affect transmission effectiveness.

All naïve contact pigs had positive counts of fluoroquinolone-resistant *E. coli *after only two days of contact, demonstrating the strong infectious pressure exerted by inoculated animals. After a 4- to 5-day decline in bacterial counts in the faeces of inoculated pigs, an overall increase was observed from day 6 until the end of the experiment. These results evidenced the possible re-infection of inoculated pigs by newly contaminated animals. This assumption is also supported by the fact that control inoculated pigs which were not mixed with naïve animals did not exhibit the same behaviour, i.e. their bacterial counts declined steadily from day 2 until the end of the experiment. Such re-infections could favour the within-group persistence of resistant bacteria.

A maximum likelihood method was used for parameter estimation which took into account the time-course of the transmission process, as the re-infections of inoculated animals precluded the use of other algorithms such as final size algorithm [24], as well as individual bacterial counts. The threshold transmission level (5*10^3 ^CFU/g) was fixed according to literature data [28] and additional specific transmission experiments would be needed to establish the infectious dose for the resistant *E. coli *strain. However, the method used in the present study is based on the classical hypotheses of the SIR epidemiological model, accounting for horizontal transmission between individuals. The roles of environmental and airborne transmissions were not considered and would need further investigation.

Three shedding-levels were considered in the present study, resulting in 3 nested models to allow for heterogeneous transmission rates. Transmission by low-shedders was indeed 2.5 times less efficient than that of high-shedders. The role of variations in individual infectiousness on the course of infection has been highlighted in several studies. Indeed, transmission rates could be influenced by several factors such as time-since-infection [16,17,32] or individual bacterial counts [33]. The role of super-spreaders was clearly identified for different infectious agents (e.g. MRSA [34], *E. coli *O157:H7 [35], *Salmonella *[36]). As transmission rates were found to be related to the individual bacterial counts, modelling the dynamics of drug-resistant bacteria within a pig herd would require representing the within-host dynamics of the bacterial population accounting for different susceptibility levels [37,38]. Such a modelling approach should take into account the resistance mechanisms of bacteria to targeted antibacterial agents (plasmidic or chromosomal genes) and the impact of selective pressure exerted by antimicrobial exposure. To this end, the within-host bacterial population model, accounting for the pharmacokinetic-pharmacodynamic aspect, could be coupled with an epidemiological model representing the spread of resistance within the population. Such a model would allow the identification of key management and treatment strategies that could limit or prevent the spread of resistant strains between individuals. The present study, focussing on the transmission of an *E. coli *strain harbouring chromosomal resistance to fluoroquinolones, could be a cornerstone for the development of this pharmaco-epidemiological model.

## Competing interests

The authors declare that they have no competing interests.

## Authors' contributions

AM analysed the data and drafted the manuscript, ALR and EJ performed bacteriological studies, RC supervised the experiment, EJ, NR, PS, ML, and CC conceived of the study, participated in its design and coordination. All authors read and approved the final manuscript.
